# Corosolic acid enhances the antitumor effects of chemotherapy on epithelial ovarian cancer by inhibiting signal transducer and activator of transcription 3 signaling

**DOI:** 10.3892/ol.2013.1591

**Published:** 2013-09-18

**Authors:** YUKIO FUJIWARA, KIYOMI TAKAISHI, JUNKO NAKAO, TSUYOSHI IKEDA, HIDETAKA KATABUCHI, MOTOHIRO TAKEYA, YOSHIHIRO KOMOHARA

**Affiliations:** 1 Department of Cell Pathology, Graduate School of Medical Sciences, Kumamoto University, Kumamoto 860-8556, Japan; 2 Department of Obstetrics and Gynecology, Graduate School of Medical Sciences, Kumamoto University, Kumamoto 860-8556, Japan; 3 Department of Natural Medicine, Faculty of Pharmaceutical Sciences, Sojo University, Kumamoto 860-0082, Japan

**Keywords:** macrophage, signal transducer and activator of transcription 3, ovarian cancer, corosolic acid

## Abstract

Resistance to chemotherapy poses a serious problem for the treatment of advanced epithelial ovarian cancer patients. The mechanisms of chemoresistance are complex and studies have implicated signal transducer and activator of transcription 3 (STAT3) signaling in the chemoresistance of cancer cells. The present study investigated whether corosolic acid (CA), which has been previously reported to be a STAT3 inhibitor, was able to increase the sensitivity to chemotherapeutic drugs in epithelial ovarian cancer cells. CA also markedly enhanced the anticancer effect of paclitaxel, cisplatin and doxorubicin. In addition, CA abrogated the cell-cell interactions between macrophages and epithelial ovarian cancer cells and inhibited the macrophage-induced activation of epithelial ovarian cancer cells. These data indicated that CA was able to reverse the chemoresistance of epithelial ovarian cancer cells and suppress the cell-cell interaction with tumorigenic macrophages. Thus, CA may be useful as an adjuvant treatment to patients with advanced ovarian and other types of cancer due to the multiple anticancer effects.

## Introduction

Epithelial ovarian cancer is the fourth most common cause of cancer-related mortality in females worldwide (1). The current treatment algorithms for newly diagnosed patients incorporate surgical cytoreduction and platinum/taxane-based chemotherapy (2,3). Despite initial response rates of ~80%, the disease recurs in 75% of cases (2,3). The recurrent cases require additional chemotherapy, but often terminate treatment due to the adverse effects of repetitive chemotherapy. Therefore, new approaches to patients with recurrent epithelial ovarian cancer are imperative.

Signal transducer and activator of transcription 3 (STAT3) is a well-known signaling molecule that is associated with cell proliferation, survival, angiogenesis and immunosuppression. The activation of STAT3 is considered to be significant for cancer progression (4). In numerous types of malignant tumors, STAT3 activation in cancer cells is associated with a poor clinical prognosis or higher grade histological malignancies (5). Novel compounds that inhibit STAT3 have been reported, a number of which are now in clinical trials for patients with malignant tumors (6). As STAT3 activation in cancer cells is known to cause resistance to chemotherapy and radiotherapy, STAT3 inhibition is considered to be effective for patients with advanced malignant tumors (6–8).

Corosolic acid (CA), a natural compound derived from apple pomace, is a potent STAT3 inhibitor and inhibits the proliferation of glioblastoma and osteosarcoma cells (9,10). Furthermore, the administration of CA has been shown to significantly suppress subcutaneous tumor development and lung metastasis in a model of osteosarcoma (10).

The present study examined whether CA has a synergistic effect with chemotherapy on epithelial ovarian cancer *in vitro,* in order to identify whether it may be beneficial in the treatment of advanced epithelial ovarian cancer.

## Materials and methods

### Cell culture

The human ovarian carcinoma SKOV3, RMG-1, and ES-2 cell lines were purchased from American Type Culture Collection (Manassas, VA, USA) and were maintained in RPMI-1640 supplemented with 10% fetal bovine serum (FBS). Peripheral blood mononuclear cells were obtained from healthy volunteer donors, who gave written informed consent for participation in this study. The study was approved by the ethics committee of Kumamoto University (Kumamoto, Japan). CD14^+^ monocytes were purified from the peripheral blood mononuclear cells by positive selection using magnetic-activated cell sorting technology (Miltenyi Biotec., Bergisch Gladbach, Germany) as described previously (11). The monocytes were cultured in Dulbecco’s modified Eagle’s medium supplemented with 10% FBS and 10 ng/ml granulocyte-macrophage colony-stimulating factor (Wako, Tokyo, Japan) for five days, and stimulated with tumor cell supernatant in order to differentiate the macrophages from the M2 phenotype.

### Extraction and isolation of CA from apple pomace

CA was isolated from the apple pomace as described previously (9). Briefly, CA was extracted with a mixed solution of MeOH and CHCl_3_ (1:1), loaded onto a Diaion HP-20 column (Mitsubishi Chemical, Tokyo, Japan) and eluted with H_2_O and MeOH. The MeOH eluate was separated using a silica gel column (Kantochemical Co. Inc., Tokyo, Japan) and eluted with a mixed solution of hexane and ethyl acetate. The CA-containing fraction was further purified using a silica gel column and eluted with a mixture of CHCl_3_ and ethyl acetate to yield pure CA.

### STAT3 activation assay

STAT3 activation was determined by measuring the increased expression of phosphorylated STAT3 by western blot analysis. The protein (10 μg) was run on a 10% sodium dodecyl sulfate-polyacrylamide gel and transferred to polyvinylidine fluoride transfer membranes (Millipore, Bedford, MA, USA). To detect the phosphorylated (phospho)-STAT3, the membranes were exposed to an anti-phospho-STAT3 antibody (D3A7, Cell Signaling, Danvers, MA, USA) and visualized by horseradish peroxidase-conjugated anti-rabbit IgG antibody (Santa Cruz Biotechnology Inc., Santa Cruz, CA, USA) with an enhanced chemiluminescence western blotting detection reagent (GE Healthcare, Tokyo, Japan). The molecular size of phospho-STAT3 that was detected by the immunoblotting procedure was ~80 kDa. To detect the STAT3 protein, the membranes were exposed to an anti-STAT3 antibody (sc-8019; Santa Cruz Biotech, Dallas, TX, USA) and visualized by horseradish peroxidase-conjugated anti-mouse IgG antibody with an ECL western blotting detection reagent. The molecular size of STAT3 that was detected by the immunoblotting procedure was ~80 kDa. These membranes were re-blotted with an anti-β-actin antibody as an internal calibration control.

### Cell proliferation and cytotoxic assay

Briefly, 1×10^4^ SKOV3, RMG-1 or ES-2 cells were cultured in 96-well plates in quadruplicate as previously described. Anticancer drugs, including CA, paclitaxel (PTX), cisplatin (CDDP) or doxorubicin (DOX) (Wako), were then added to the cells. The cell viability was determined using a WST assay (WST-8 cell counting kit; Dojin Chemical, Kumamoto, Japan) according to the manufacturer’s instructions. In order to analyze the cytotoxic activity, the amount of lactate dehydrogenase (LDH) that was released into the culture supernatants was calculated using an LDH release assay (LDH-cytotoxic test kit; Wako).

### Assessment of apoptosis

The apoptotic cells in the sections were detected by the terminal deoxynucleotidyl transferase (TdT)-mediated dUTP nick end-labeling (TUNEL) technique using an ApopTag Peroxidase In Situ Apoptosis Detection kit (Intergen Co., Purchase, NY, USA). To visualize the reaction, anti-digoxigenin-peroxidase was applied for 30 min at room temperature. For the negative controls, distilled water was used instead of the TdT enzyme.

### Immunohistochemistry

The co-culture cells were fixed in 10% neutral buffered formalin and embedded in paraffin wax. Deparaffinized sections were immersed in 0.3% hydrogen peroxide solution and treated with anti-BrdU (Abbiotec, San Diego, CA, USA) and anti-pSTAT3 (Cell Signaling Technology, Tokyo, Japan) antibodies. The sections were subsequently treated with a HRP-conjugated secondary antibody (Nichieri Bioscience, Tokyo, Japan). Reactions were visualized with diaminobenzidine. The number of BrdU-positive cells were counted among 200 randomly selected tumor cells under a microscope.

### Statistical analysis

All data are representative of two or three independent experiments that were performed in quadruplicate. The data are expressed as the mean ± standard deviation. The Mann-Whitney U test was used for the two-group comparison. P<0.05 was considered to indicate a statistically significant difference.

## Results

### CA inhibits epithelial ovarian cancer cell proliferation by suppressing STAT3 activation

**Figure 1:**
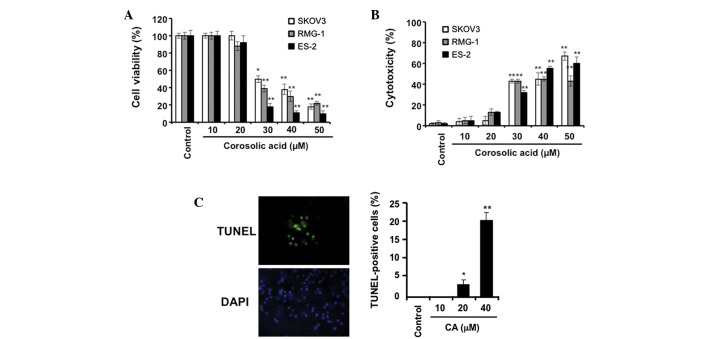
Effect of CA on the proliferation of ovarian carcinoma cells. The ovarian carcinoma cells (SKOV3, RMG-1, and ES-2) were incubated with the indicated concentrations of CA for 48 h, followed by (A) determination of cell viability and (B) cell cytotoxicity, by WST-8 assay and LDH assay, respectively (as described in Materials and methods). (C) The ES-2 cells were incubated with the indicated concentrations of CA for 5 h, followed by the determination of cell apoptosis by TUNEL staining (as described in Materials and methods). Data are presented as the mean ± SD. ^*^P<0.01 and ^**^P<0.001 vs. the control. CA, corosolic acid; DAPI, 4′,6-diamidino-2-phenylindole; TUNEL, terminal deoxynucleotidyl transferase-mediated dUTP nick end-labeling; LDH, lactate dehydrogenase.

**Figure 2:**
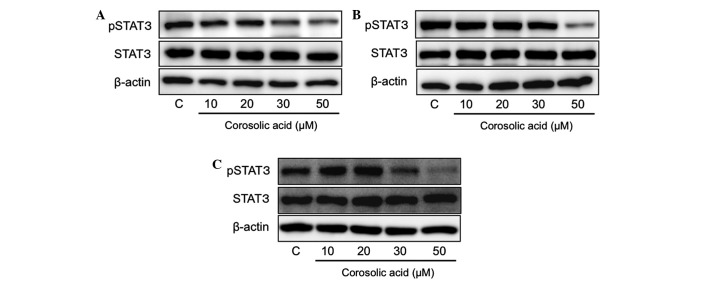
Effect of CA on STAT3 activation of ovarian carcinoma cells. Ovarian carcinoma cells, (A) SKOV3, (B) RMG-1 and (C) ES-2, were incubated with the indicated concentrations of CA for 24 h, followed by determination of pSTAT3, STAT3 and β-actin expression by western blot analysis (as described in Materials and methods). CA, corosolic acid; STAT3, signal transducer and activator of transcription 3; pSTAT3, phosphorylated signal transducer and activator of transcription 3.

The effect of CA on the proliferation of epithelial ovarian cancer cells was measured. CA was observed to inhibit the proliferation of the SKOV3, RMG-1 and ES-2 cells at a concentration of at least 30 μM (Fig. 1A and B). Following this, it was investigated whether CA caused cancer cell apoptosis using a TUNEL assay. CA induced cell apoptosis in the SKOV3, RMG-1 and ES-2 cells in the preliminary examination. As shown in Fig. 1C, CA was clearly observed to induce apoptosis in the ES-2 cells in the main examination. The effect of CA on STAT3 activation was examined and it was demonstrated that CA inhibited STAT3 activation at a concentration of at least 30 μM in the epithelial ovarian cancer cells (Fig. 2).

### CA increases the sensitivity of epithelial ovarian cancer cells to anticancer drugs

**Figure 3:**
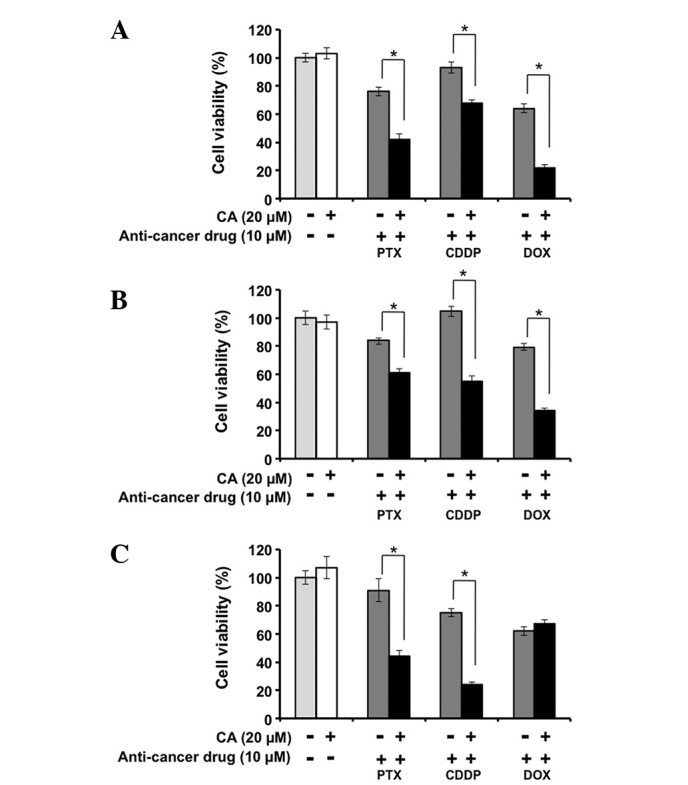
Combined effect of CA and anticancer drugs on the proliferation of ovarian carcinoma cells. Ovarian carcinoma cells, (A) SKOV3, (B) RMG-1 and (C) ES-2, were incubated with 10 μM anticancer drugs, PTX, CDDP and DOX, concurrently with or without 20 μM CA for 24 h. Cell viability was then determined using a WST-8 assay (as described in Materials and methods). Data are presented as the mean ± SD. ^*^P<0.01 vs. the control. CA, corosolic acid; PTX, paclitaxel; CDDP, cisplatin; DOX: doxorubicin.

**Figure 4:**
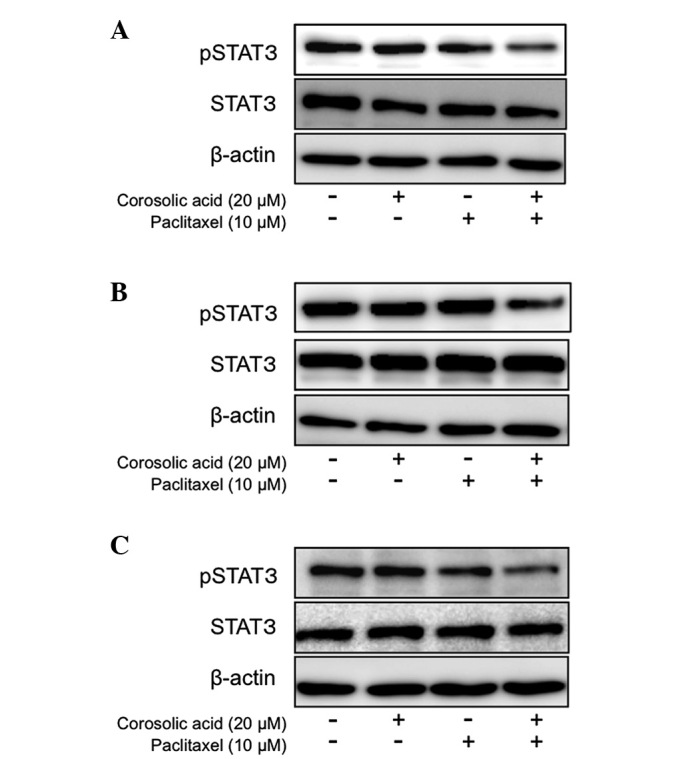
Combinational effect of CA and anticancer drugs on STAT3 activation of ovarian carcinoma cells. The ovarian carcinoma cells, (A) SKOV3, (B) RMG-1 and (C) ES-2, were incubated with 20 μM CA and/or 10 μM paclitaxel for 24 h, followed by determination of pSTAT3, STAT3 and β-actin expression by western blot analysis (as described in Materials and methods). CA, corosolic acid; STAT3, signal transducer and activator of transcription 3; pSTAT3, phosphorylated signal transducer and activator of transcription 3.

To elucidate whether CA enhances the anticancer activity of the anticancer drugs in the epithelial ovarian cancer cells, the combinational effects of CA and the anticancer drugs, including PTX, CDDP and DOX, was examined. As CA demonstrated no anticancer effects at a concentration of 20 μM, three epithelial ovarian cancer cell lines (SKOV3, RMG-1 and ES-2) were incubated with 20 μM CA for 24 h, concurrently with an incubation of 10 μM anticancer drugs. As shown in Fig. 3A, the cell viability was not changed by stimulation with 20 μM CA alone in the SKOV3 cells. By contrast, 20 μM CA enhanced the inhibitory effect of the anticancer drugs on the proliferation of the SKOV3 cells. Similar results were observed in the RMG-1 and ES-2 cells (Fig. 3B and 3C). These results demonstrate that CA enhances the anticancer activity of anticancer drugs in epithelial ovarian carcinoma cells. Notably, the combination of 20 μM CA and PTX inhibited STAT3 activity in the epithelial ovarian cancer cells (Fig. 4), though CA alone or PTX alone had lesser effects on the STAT3 activity (Fig. 4). These findings suggest that CA enhances the inhibitory effects of anticancer drugs by STAT3 inhibition.

### CA inhibits macrophage polarization into the M2 phenotype, which induces cancer cell proliferation

**Figure 5:**
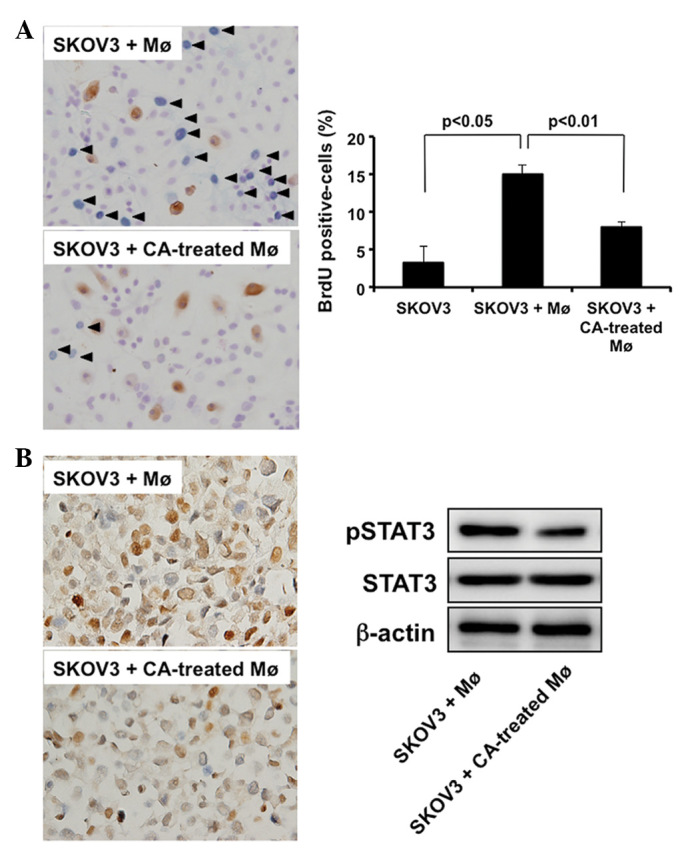
Effect of CA-treated macrophages on STAT3 activation and cell proliferation in epithelial ovarian carcinoma. (A) HMDM were treated with 20 μM CA for 24 h and the SKOV3 cells were incubated with CA-treated macrophages for 24 h, followed by determination of BrdU-positive cells by immunohistochemistry (as described in Materials and methods). (B) HMDM were treated with 20 μM CA for 24 h, while the SKOV3 cells were incubated with CA-treated macrophages for 24 h, followed by determination of STAT3 activation by immunohistochemistry (left) and western blot analysis (right) (as described in Materials and methods). CA, corosolic acid; STAT3, signal transducer and activator of transcription 3; HMDM, human monocyte-derived macrophages; Mø, macrophage.

Direct cell-cell interactions between M2 macrophages and epithelial ovarian cancer cells have previously been reported to induce STAT3 activation and a tumorigenic microenvironment in the ascites fluid of advanced epithelial ovarian cancer patients (12,13). Furthermore, CA has previously been observed to significantly inhibit M2 polarization of macrophages (9). Therefore, the present study examined the effect of CA-treated macrophages on STAT3 activation and cell proliferation in the SKOV3 cells. As shown in Fig. 5A, BrdU incorporation in the SKOV3 cells was strongly increased by coculture with the M2 macrophages, whereas BrdU incorporation did not differ following coculture with the CA-treated macrophages. STAT3 activation was also lower in the SKOV3 cells that were cocultured with the CA-treated macrophages than in the SKOV3 cells that were cocultured with the M2 macrophages (Fig. 5B).

## Discussion

The present study demonstrated that the anticancer effects of CA on epithelial ovarian cancer cells are due to its suppressive effect on STAT3. Furthermore, CA enhanced the anticancer effect of chemotherapeutic agents. STAT3 activation is a well-known signal that is associated with cell survival or resistance to apoptosis and this effect is considered to be induced by the upregulation of anti-apoptotic genes, including Bcl-X, MCL-1 and survivin (6). CA is suggested to downregulate these anti-apoptotic genes in epithelial ovarian cancer cells (6).

Numerous tumor-associated macrophages (TAMs) are detected in the cancer tissues (14–16) and ascite fluid of patients with advanced epithelial ovarian cancer. Almost all TAMs in epithelial ovarian cancer and ascites have shown to be polarized to the M2 anti-inflammatory phenotype (12,13). *In vitro* coculture experiments have demonstrated that STAT3 activation in epithelial ovarian cancer cells was strongly induced by coculturing with M2 macrophages and was only slightly induced by coculturing with M1 macrophages (12). A similar phenomenon has been observed in glioma and lymphoma cells (17,18). In the present study, coculture experiments were performed, which demonstrated that CA suppressed STAT3 activation and BrdU incorporation in epithelial ovarian cancer cells by abrogating macrophage differentiation into the M2 phenotype.

In conclusion, the *in vitro* efficacy of CA for the treatment of advanced epithelial ovarian cancer was demonstrated in this study. CA inhibited cancer cell proliferation and enhanced chemosensitivity by suppressing STAT3 activation. CA also abrogated macrophage differentiation into the M2 phenotype and cancer cell activation due to cell-cell interaction with macrophages. As STAT3 activation leads to cancer progression in other types of malignant tumors, including glioma and kidney cancer, and as M2 TAMs are also associated with cancer development in numerous kinds of malignant tumors, CA may also be useful as an adjunctive treatment for patients with advanced malignant tumors other than epithelial ovarian cancer.
